# Dual Oxidase Maturation Factor 1 Positively Regulates RANKL-Induced Osteoclastogenesis via Activating Reactive Oxygen Species and TRAF6-Mediated Signaling

**DOI:** 10.3390/ijms21176416

**Published:** 2020-09-03

**Authors:** Yoon-Hee Cheon, Chang Hoon Lee, Da Hye Jeong, Sung Chul Kwak, Soojin Kim, Myeung Su Lee, Ju-Young Kim

**Affiliations:** 1 Core Research Facility Center, School of Medicine, Wonkwang University, Iksan 54538, Korea; hanleuni@naver.com; 2 Musculoskeletal and Immune Disease Research Institute, School of Medicine, Wonkwang University, Iksan 54538, Korea; lck110@wku.ac.kr (C.H.L.); jdah1017@naver.com (D.H.J.); albireo0127@naver.com (S.K.); 3 Division of Rheumatology, Department of Internal Medicine, Wonkwang University Hospital, Iksan 54538, Korea; 4 Department of Anatomy, School of Medicine, Wonkwang University, Iksan 54538, Korea; ksc960@naver.com; 5 Medical Convergence Research Center, Wonkwang University Hospital, Iksan 54538, Korea

**Keywords:** Duoxa1, osteoclast, RANKL, ROS, TRAF6

## Abstract

Receptor activator of NF-κB ligand (RANKL) induces generation of intracellular reactive oxygen species (ROS), which act as second messengers in RANKL-mediated osteoclastogenesis. Dual oxidase maturation factor 1 (Duoxa1) has been associated with the maturation of ROS-generating enzymes including dual oxidases (Duox1 and Duox2). In the progression of osteoclast differentiation, we identified that only Duoxa1 showed an effective change upon RANKL stimulation, but not Duox1, Duox2, and Duoxa2. Therefore, we hypothesized that Duoxa1 could independently act as a second messenger for RANKL stimulation and regulate ROS production during osteoclastogenesis. Duoxa1 gradually increased during RANKL-induced osteoclastogenesis. Using siRNA or retrovirus transduction, we found that Duoxa1 regulated RANKL-stimulated osteoclast formation and bone resorption positively. Furthermore, knockdown of Duoxa1 decreased the RANKL-induced ROS production. During Duoxa1-related control of osteoclastogenesis, activation of tumor necrosis factor receptor-associated factor 6 (TRAF6)-mediated early signaling molecules including MAPKs, Akt, IκB, Btk, Src and PLCγ2 was affected, which sequentially modified the mRNA or protein expression levels of key transcription factors in osteoclast differentiation, such as c-Fos and NFATc1, as well as mRNA expression of osteoclast-specific markers. Overall, our data indicate that Duoxa1 plays a crucial role in osteoclastogenesis via regulating RANKL-induced intracellular ROS production and activating TRAF6-mediated signaling.

## 1. Introduction

Reactive oxygen species (ROS) are well known to be composed of superoxide anions, hydrogen peroxide (H_2_O_2_) and hydrogen radical containing oxygen, and are normal products of cellular metabolism. ROS are continuously produced during the metabolic processes in the body and are closely related to the prevalence of various diseases, as they act as important physiological regulators of intracellular signaling pathways and gene expression [1]. ROS are generated by the action of nicotinamide adenine dinucleotide phosphate (NADPH) oxidases and the mitochondrial electron transport process [2]. ROS overproduction by excessive stimulation of NADPH results in oxidative stress; the elevated ROS levels can cause oxidative DNA, RNA, proteins, and lipids, and the increased ROS activity can affect the pathophysiology of cancer, aging, diabetes, atherosclerosis, rheumatoid arthritis, or other diseases [3]. According to recent studies related to ROS in bone metabolism, ROS production is a key factor of bone cell function and this elevated oxidative stress influences in bone homeostasis [4,5,6].

Regulation of homeostasis in bone metabolism can rely upon the reciprocal cooperation between bone resorption by osteoclasts and bone formation by osteoblasts. Abnormal reduction in the bone mass by excess osteoclasts has been reported in bone-related diseases such as bone destruction, fracture, rheumatoid arthritis, and osteoporosis [7]. Osteoclasts differentiate from bone marrow macrophages (BMMs) derived from the monocyte/macrophage cell lineage in bone marrow cells (BMCs) [8]. Osteoclasts formed by the modulation of receptor activator of nuclear factor-κB ligand (RANKL) and macrophage colony-stimulating factor (M-CSF) for their survival, proliferation, differentiation, and activation. RANKL-induced osteoclast differentiation is subject to recruitment of tumor necrosis factor receptor -associated factor 6 (TRAF6), mitogen-activated protein kinases (MAPKs), and activator protein-1 (AP-1) [9]. In particular, nuclear factor-activated T cells c1 (NFATc1) is a master transcription factor of osteoclastogenesis, and ultimately controls bone resorption and function via regulating many osteoclast-specific markers including *cathepsin K* (*CtsK*), *osteoclast-associated receptor* (*OSCAR*), and *v-ATPase subunit d2* (*ATP6v0d2*) [9,10,11,12]. RANKL also induces the generation of ROS, causing oxidative stress during osteoclastogenesis in BMMs, and this generated ROS is involved in the signaling of TRAF6, MAPKs, and NADPH oxidases (Nox isoforms) [6,10]. Therefore, the enzymes involved in H_2_O_2_ production may exert a strong influence on osteoclast differentiation in bone.

The Nox family comprises seven isoforms: Nox1-5, dual oxidase 1 (Duox1), and dual oxidase 2 (Duox2). These are ubiquitously identified in various cell types including the epithelium and endothelium but they have general distribution patterns of H_2_O_2_-production and expressions [13]. Interestingly, several tissues were found to express dual oxidase maturation factor 1 (Duoxa1) and relatively elevated levels of Duoxa1 protein were specifically found in several tissues including the thyroid gland, respiratory system, salivary gland, and brain [13,14,15]. Duoxa1 and Duoxa2 form functional heterodimeric complexes with each Duox1 and Duox2, respectively, and these complex formations need the translocation of the elements to the plasma membrane to transmit their oxidase activity [3]. In the absence of Duoxa1, Duox cannot produce H_2_O_2_ in the thyroid and lung [16,17]. Duoxa1 is known to function as a maturation factor for Duox1; however, since Duox1 fails to appear in microglia, it may have an undetected function in mouse microglia [14]. Another report indicated that overexpression of Duoxa1 in muscle stem cells induces apoptosis and suppresses myogenesis through the increased ROS generation and a mechanism involving Duox1 and apoptosis signal-regulating kinase 1 [13].

Among the seven Nox family members, Nox1, Nox2, and Nox4 have been identified to be implicated in osteoclast differentiation [18,19,20]. Knockdown of Nox1 in BMM cells resulted in a decrease in RANKL-mediated ROS generation and osteoclastogenesis. Nox2^−/−^ and Nox4^−/−^ knock out mice each exhibited higher bone density and decreased numbers of osteoclasts [19,20]. These reports indicated that Nox family members control bone homeostasis via the regulation of ROS levels. However, the role of Duox and/or Duoxa in bone metabolism remains unclear.

To elucidate the regulatory mechanism of Duox and/or Duoxa enzymes related to H_2_O_2_ production in osteoclast differentiation, we performed quantitative real-time RT-PCR (qRT-PCR) analysis in RANKL-induced BMMs. We found that Duoxa1 was significantly upregulated in RANKL-induced BMMs, whereas Duox1, Duox2 and Duoxa2 expression was not; furthermore, the elevated Duoxa1 level led to increased ROS levels. We also performed various in vitro studies using gain and loss of Duoxa1 function.

## 2. Results

### 2.1. Knockdown of Duoxa1 Inhibits RANKL-Mediated Osteoclast Differentiation and Bone Resorbing Function

**Figure 1:**
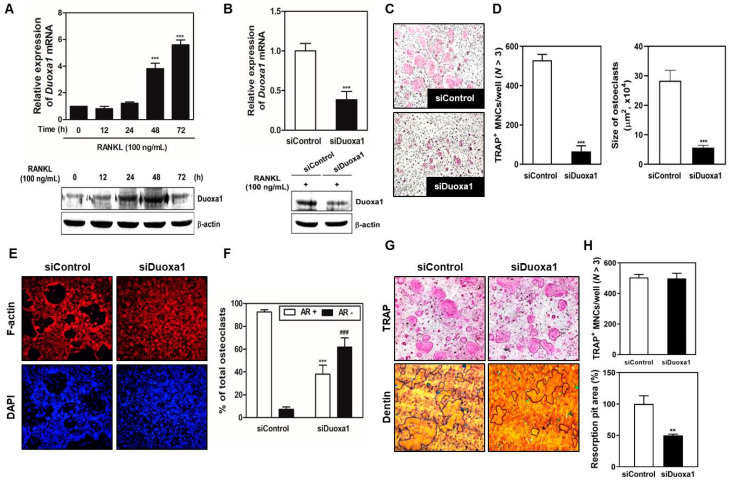
Expression pattern of Duoxa1 during osteoclastogenesis and the effect of Duoxa1 knockdown in RANKL-induced osteoclast differentiation and function. (**A**) BMMs were cultured in the presence of M-CSF (30 ng/mL) and RANKL (100 ng/mL) for the indicated time periods, and the expression of Duoxa1 was analyzed by qRT-PCR and Western blot analysis. The mRNA and protein expression were normalized to *GAPDH* mRNA and β-actin as an internal control, respectively. Data are presented as the mean ± SD of three independent experiments. *** *p* < 0.001 versus the control. (**B**–**F**) BMMs were transfected with siControl or siDuoxa1, and cultured in the presence of M-CSF (30 ng/mL) and RANKL (100 ng/mL) for 3 days. (**B**) Knockdown efficiency of Duoxa1 was confirmed by qRT-PCR and Western blot analysis. (**C**) TRAP-positive cells were determined by TRAP staining (×10). (**D**) The number of multinucleated cells (MNCs) with 3 or more nuclei (*left*) and the size of each osteoclast (µm^2^) (*right*) were counting and assessed, respectively. *** *p* < 0.001 versus the siControl. (**E**,**F**) Actin ring formation was identified by rhodamine-conjugated phalloidin staining. DAPI staining of the nucleus indicates the control (×10). (**F**) The graph was denoted as the quantification of percentage of osteoclasts having actin ring (AR+) or actin clusters (AR−). *** *p* < 0.001 versus AR^+^ of the siControl; ^###^ *p* < 0.001 versus AR− of the siControl. (**G**) Matured osteoclasts were co-cultured and formed between osteoblast cells and BMCs. The siRNAs transfected into the matured osteoclast cells after plating on dentin slices and cultured for 48 h. The bone resorption regions were highlighted by black borders (×10). (**H**) The number of TRAP-positive MNCs was counted (*upper*) and the resorption areas on dentine slices were quantified with Image J (*lower*). Data are presented as the mean ± SD of three independent experiments. ** *p* < 0.01 versus the siControl. ns = not significant.

To discover the role of ROS-producing genes involved in RANKL-mediated osteoclast differentiation, we confirmed the mRNA expression levels of Duox genes (Duox1 and Duox2) and Duox maturation factors (Duoxa1 and Duoxa2) in BMMs by qRT-PCR. Interestingly, only Duoxa1 among them was greatly increased by RANKL stimulation in BMMs (Figure 1A). All three genes except Duoxa1 did not show a significant change in mRNA expression by RANKL stimulation (data not shown). To define the role of Duoxa1 during osteoclastogenesis, we designed siRNA oligonucleotides against Duoxa1. It was confirmed that the expression of Duoxa1 was effectively knocked down by siDuoxa1 in BMM (Figure 1B) without toxicity (Figure S1). Knockdown of Duoxa1 significantly inhibited the formation of osteoclasts (Figure 1C,D) and actin ring compared to the siControl (Figure 1E,F). Moreover, the bone resorbing function of matured osteoclasts on dentin slices was significantly reduced upon Duoxa1 knockdown compared to control siRNA without affecting the survival of osteoclasts (Figure 1G,H). These results suggest that Duoxa1 plays an important role in the regulation of osteoclastogenesis.

### 2.2. Knockdown of Duoxa1 Leads to a Decrease in TRAF6-Mediated Signals

**Figure 2:**
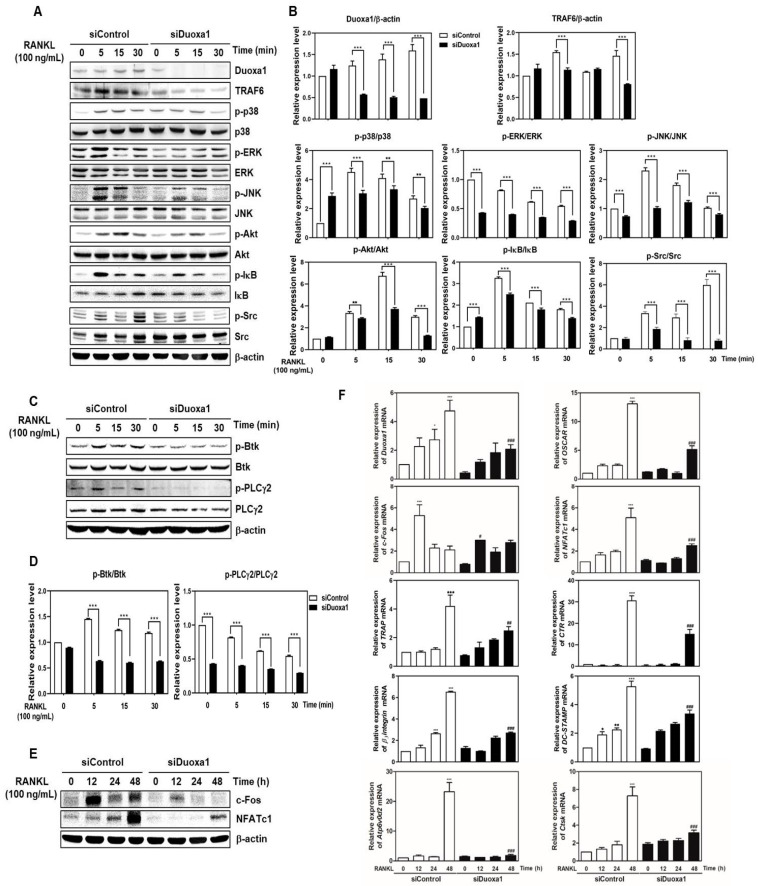
Knockdown of Duoxa1 suppresses c-Fos and NFATc1 expression accompanied by regulating the TRAF6-mediated signal pathway. (**A**–**E**) BMMs transfected with siControl or siDuoxa1 were stimulated with RANKL (100 ng/mL) for the indicated time points. Whole cell extracts were probed with the indicated antibodies. β-actin was used as an internal control. The graph indicates the phosphoprotein-to-total protein rations of each molecule or the TRAF6 to β-actin. Quantification of relative ratio of band intensity was performed using Image J software. The phosphoprotein-to-total protein or the TRAF6 ratios (relative expression levels) are represented as the mean ± standard deviation. ** *p* < 0.01, *** *p* < 0.001 versus siControl at each time. (**F**) BMMs transfected with siControl or siDuoxa1 were stimulated with RANKL (100 ng/mL) for the indicated time periods. The mRNA expression of the indicated genes was analyzed by qRT-PCR. Data are presented as the mean ± SD of three independent experiments. * *p* < 0.05; *** *p* < 0.001 versus the siControl at 0 h; ^#^ *p* < 0.05, ^##^ *p* < 0.01, ^###^ *p* < 0.001 versus siControl at each time.

Interaction between RANK and RANKL leads to the recruitment of TRAF6 to Akt, MAPKs, Src and the NF-κB pathway. RANK and ITAM-mediated interaction are also involved in synergistic signaling in osteoclast-associated mechanisms, which activate the Btk-PLCγ2 pathway [21]. In this part, we examined whether Duoxa1 regulates the signaling pathway associated with osteoclasts using siDuoxa1. RANKL-stimulated the expression of TRAF6 and the phosphorylation of Akt, IκB, Src and MAPKs (p38, ERK, and JNK) were distinctly decreased upon knockdown of Duoxa1 compared to the siControl (Figure 2A,B). Furthermore, increased activation of Btk-PLCγ2 signaling after RANKL treatment was attenuated by siDuoxa1 compared to siControl (Figure 2C,D). Treatment of RANKL increased the c-Fos and NFATc1 mRNA and protein levels, whereas silencing of Duoxa1 significantly suppressed the RANKL-mediated induction of both transcription factors (Figure 2E,F). RANKL also increased *OSCAR, TRAP, calcitonin receptor* (*CTR*)*, β_3_-integrin, DC-STAMP, Atp6v0d2*, and *CtsK* mRNA expression, whereas silencing of Duoxa1 suppressed these activities (Figure 2F). These data show that knockdown of Duoxa1 suppresses RANKL–RANK-mediated signaling in osteoclastogenesis.

### 2.3. Overexpression of Duoxa1 Plays a Positive Role in RANKL-Mediated Osteoclast Differentiation

**Figure 3:**
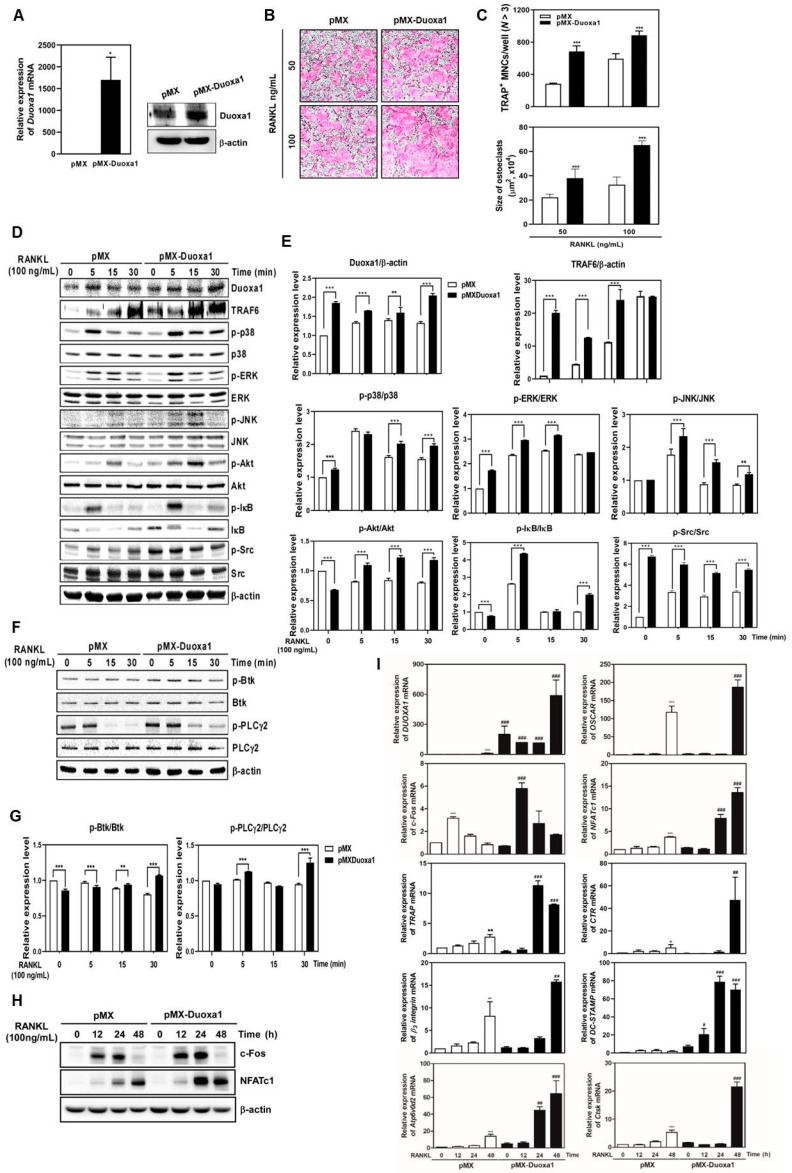
Overexpression of Duoxa1 enhances osteoclast differentiation by regulating the TRAF6-mediated signal molecules and osteoclast marker genes. (**A**) BMMs were transduced with the pMX or pMX-Duoxa1 retrovirus. Overexpressed *Duoxa1* mRNA levels were observed by qRT-PCR and western blot analysis. * *p* < 0.05 versus the pMX. (**B**,**C**) BMMs were transduced with the pMX or pMX-Duoxa1 retrovirus and then incubated with M-CSF (30 ng/mL) and RANKL (50 or 100 ng/mL) for 3 days. (**B**) Osteoclasts were stained for TRAP (×10). (**C**) TRAP-positive MNCs with 3 or more nuclei were counted (*upper*) and the size of each osteoclast was assessed (*lower*). *** *p* < 0.001 versus the pMX. (**D**–**H**) BMMs transfected with pMX or pMX-Duoxa1 were stimulated with RANKL (100 ng/mL) for the indicated time points. Whole cell extracts were probed with the indicated antibodies. β-actin was used as an internal control. The graph indicates the phosphoprotein-to-total protein rations of each molecule or the TRAF6 to β-actin. Quantification of relative ratio of band intensity was performed using Image J software. The phosphoprotein-to-total protein or the TRAF6 ratios (relative expression levels) are represented as the mean ± standard deviation. ** *p* < 0.01, *** *p* < 0.001 versus siControl at each time. (**I**) BMMs transfected with pMX or pMX-Duoxa1 were stimulated with RANKL (100 ng/mL) for the indicated time periods. The mRNA expression of indicated genes was analyzed by qRT-PCR. Data are presented as the mean ± SD of three independent experiments. ^*^p < 0.05, ** *p* < 0.01, *** *p* < 0.001 versus the pMX at 0 h; ^#^ *p* < 0.05, ^##^ *p* < 0.01, ^###^ *p* < 0.001 versus the pMX at each time.

Compared with the functional role of Duoxa1 gene using siDuoxa1 in osteoclast differentiation, we next demonstrated the overexpression effect of Duoxa1 using retroviral transduction. Overexpression of Duoxa1 by transduction of pMX-Duoxa1 was significantly increased after RANKL treatment (Figure 3A), and this overexpression of Duoxa1 accelerated the formation of TRAP-positive MNCs (Figure 3B,C). As a result, it turned out that this positive effect was supported by the upregulation of c-Fos and NFATc1 at both the mRNA (Figure 3I) and protein (Figure 3H) levels, and their upstream signal transducers, including TRAF6, p38, ERK, JNK, Akt, IκB, Src, Btk, and PLCγ2 (Figure 3D–G). In addition, the positive regulation of Duoxa1 on c-Fos and NFATc1 contributed to the increase in the expression of various osteoclast-associated marker genes, such as *OSCAR*, *TRAP*, *CTR, β_3_-integrin*, *DC-STAMP, Atp6v0d2*, and *CtsK* (Figure 3I). These results show that Duoxa1 acts as a positive regulator in RANKL-mediated osteoclastogenesis via the activation of TRAF6, p38, ERK, JNK, Akt, IκB, Src, Btk, and PLCγ2 signal pathways, followed by the induction of c-Fos and NFATc1, leading to decreased levels of osteoclast-specific genes.

### 2.4. Knockdown of Duoxa1 Attenuates RANKL-Induced ROS Generation

**Figure 4:**
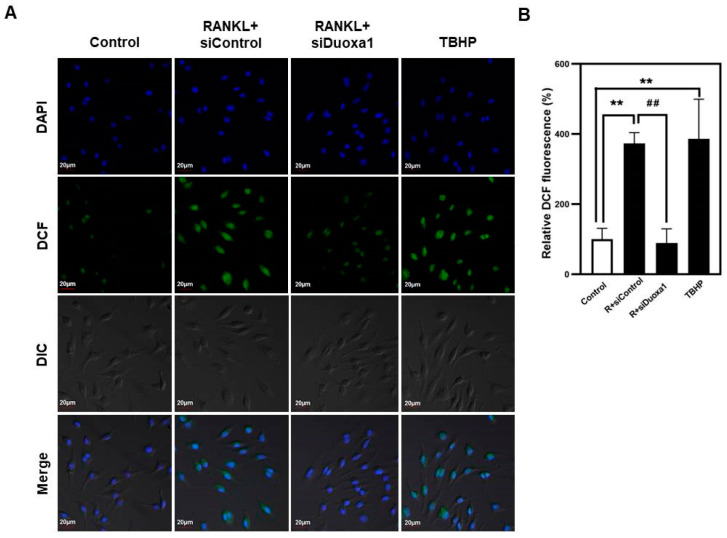
Knockdown of Duoxa1 inhibits RANKL-induced ROS production. (**A**) BMMs transfected with siControl or siDuoxa1 were treated with RANKL for 10 min, and ROS levels were determined by DCF fluorescence detection using a confocal laser-scanning microscope (×20). (**B**) The DCF fluorescence intensity was confirmed for the region of interest (ROI). Results are representative of at least 3 independent sets of similar experiments. TBHP was used as the standard control for ROS production. Data are presented as the mean ± SD of three independent experiments. ** *p* < 0.01 versus the negative siControl. ^##^ *p* < 0.01 versus the positive siControl.

We finally examined the influence of Duoxa1 in RANKL-stimulated intracellular ROS accumulation. Intracellular production of ROS in osteoclast precursor cells was measured using the cell-permeant oxidative-sensitive DCF fluorescence intensity by confocal laser-scanning microscopy. The addition of RANKL to BMMs induced ROS production in a manner similar to TBHP as the standard control of ROS production. However, ROS levels enhanced by RANKL were markedly inhibited by siDuoxa1 (Figure 4A,B). Contrary to these results, ROS production increased by RANKL was significantly enhanced by overexpression of Duoxa1 by transduction of pMX-Duoxa1 (Figure S2). These data indicate that Duoxa1 plays an important role in the ROS generation of osteoclast precursor cells and siDuoxa1 suppresses osteoclastogenesis via inhibiting ROS production.

## 3. Discussion

Duoxa1 is generally known as an organizing subunit for the translocation and surface expression of the Duox enzymes which directly produce H_2_O_2_ [15]. Duoxa1 is involved in the maturation and translocation of Duox1 from the cytosol to plasma membrane to produce a mature and completely functional enzyme [16,17]. However, in addition to activating Duox1, Duoxa1 may have other unknown functions, as confirmed by the high levels of Duoxa1 protein in several adult tissues including muscle satellite cells, the thyroid and salivary glands, and lungs [13,14,15]. In the study by Sandiford et al., overexpression of Duoxa1 was detected in primary myoblasts and the high level of Duoxa1 throughout myogenic differentiation resulted in increased H_2_O_2_ formation and defective myoblast fusion [13]. According to Serendenina et al., Duoxa1 is upregulated in spinal cord tissue with the disease course against Duox1 that shows expression below detection levels in tissues. High Duoxa1 expression was detected in the microglia, resident immune cells of the central nerve system, in contrast to Duox1 absence, and may have a presently unknown function in microglia [14]. Stanislas et al. also mentioned the possibility that Duoxa1 may function independently of Duox1 in ROS-producing mechanisms [15]. Similarly, we found that Duoxa1 was upregulated in RANKL-stimulated osteoclast differentiation in proportion to time (Figure 1A). However, Duox1 was below the detection levels in RANKL-induced BMMs. This means that Duoxa1 may directly engage in ROS generation, which may promote osteoclast differentiation. In other words, Duoxa1 may function independently of Duox1 in osteoclast differentiation and ROS production in bone. Our study is the first to emphasize the importance of Duoxa1 in osteoclast differentiation through the control of ROS generation. These abilities were confirmed by subsequent observations that siDuoxa1 in RANKL-induced BMMs inhibited osteoclast formation and function (Figure 1) and overexpression of Duoxa1 inhibited osteoclastogenesis (Figure 3) by regulating ROS production (Figure 4).

Bone homeostasis and mineral remodeling requires complex communication between bone-resorbing osteoclasts and bone-forming osteoblasts in bone [6,7,8,9]. In many pathological conditions, bone resorption of osteoclasts exceeds the formation of osteoblasts, leading to excessive bone destruction. Osteoclasts are derived from the monocyte/macrophage lineage of hematopoietic stem cells, and osteoclasts differentiate under M-CSF and RANKL treatment [11]. The binding of the M-CSF to c-Fms provides signals required for proliferation and survival of osteoclast precursor cells, whereas binding of RANK and RANKL leads to ROS production and recruitment of TRAF6, and upon stimulation, the precursors fuse and form multinucleate osteoclasts. Progressive accumulation of ROS by RANKL stimulation modulates the expression of several ROS-related genes including TRAF6, Rac1, and Nox isoforms [6,7,8,9,10]. The expression of Duoxa1 increases during the process of RANKL-induced osteoclastogenesis (Figure 1A). Indeed, siDuoxa1 resulted in the inhibition of both RANKL-induced ROS production (Figure 4) and osteoclast differentiation and bone resorbing functions (Figure 1C–H). Further overexpression of Duoxa1 abundantly improved RANKL-induced multinuclear cells from BMMs (Figure 3).

RANK and RANKL-mediated osteoclastogenesis and bone resorbing function lead to TRAF6 recruitment to activate MAPKs (p38, ERK, and JNK), c-Fos, activator protein-1 (AP-1) and NF-κB, all of which are required for the induction of NFATc1 [10,11,22]. RANKL stimuli are also dependent on the generation of Ca^2+^ signaling through the ITAM-mediated signal cascade that activates Btk-PLCγ2 calcium signaling to interact with NFATc1 [21]. Notably, we found that siDuoxa1 transfection inhibited the activation of TRAF6 and calcium signal recruitment by RANKL through the Akt/MAPKs/NF-κB/c-Src and Btk-PLCγ2 signal pathways (Figure 2A–D). In contrast, upregulated Duoxa1 in pMX-Duoxa1 retrovirus-infected BMMs augmented the activation of TRAF6 and calcium signal recruitment by RANKL (Figure 3D–G). Owing to the known roles of ROS in osteoclastogenesis and signaling pathways, we identified whether Duoxa1 could alter RANKL-induced signaling by regulating ROS generation during osteoclastogenesis. We observed that siDuoxa1 markedly inhibited the RANKL-induced intracellular ROS production measured by a cell-permeant oxidation-sensitive dye, DCFH-DA (Figure 4A,B). These results indicate that Duoxa1 regulates osteoclastogenesis by activating RANKL-induced ROS production and TRAF6/Btk-PLCγ2 signal pathways.

The adhesion molecules β_3_-integrin and c-Src play important roles in regulating the bone resorption of osteoclasts by mediating their migration and adhesion activities [23,24]. It has also been reported that osteoclasts deficient for c-Src exhibited reduced motility and abnormal organization in the ruffle border and that they lacked the cytoskeletal elements necessary for bone resorption [23]. Therefore, c-Src is required for osteoclastic bone resorption [25]. CtsK and CTR degrade the organic bone matrix and contribute to bone resorptive activity [11,12]. RANKL-induced ROS generation and TRAF6-MAPK, NF-kB, c-Src/Btk-PLCγ2 signal leads to activation of NFATc1 and osteoclast differentiation for bone resorption [10]. The master transcription factor NFATc1 affects the expression of osteoclast-specific genes including *OSCAR, TRAP, CTR, β_3_-integrin, DC-STAMP, ATP6v0d2*, and *CtsK*, which are related to formation of multinuclear cells by fusion of osteoclasts [11,12]. In this study, knockdown of Duoxa1 significantly inhibited the expression of transcription factors and marker genes (Figure 2E,F), whereas overexpression of Duoxa1 reversed the expression of these genes (Figure 3H,I). Moreover, RANKL exerted anti-apoptotic effects in mature osteoclasts, which apparently increased bone-degradative activity and accelerated bone resorption. In our study, siDuoxa1 suppressed actin polymerization (Figure 1E,F) and bone resorption by matured osteoclasts (Figure 1G,H). Thus, these results show the disruption of osteoclastic cytoskeleton by siDuoxa1, which abolishes the bone resorbing activity by matured osteoclasts in vitro.

In conclusion, these findings highlight the crucial role of ROS generation by Duoxa1 in osteoclast differentiation and reveal Duoxa1 as a potential therapeutic target in treating bone diseases associated with excess osteoclasts-induced bone loss such as osteoporosis and Paget’s disease of bone.

## 4. Materials and Methods

### 4.1. Chemicals, Reagents and Constructs

Full-length wild-type Duoxa1 was amplified using PCR from mouse cDNA and ligated into the pMX-IRES-EGFP vector as pMX-Duoxa1 using the BamHI and XhoI (Enzynomics, Daejeon, Korea) sites. The following primers were used: *Duoxa1*-For, 5′-GCTAGGATCCATGGCTGCTCTTGGACACAC-3′ and *Duoxa1*-Rev, 5′-CGACTCGAGCAGGGAACAGTCGGACTCTTTG-3′. TRIzol reagent was obtained from Life Technologies (Carlsbad, CA, USA). A monoclonal β-actin (A5441) antibody and DAPI (D9542) were obtained from Sigma (St. Louis, MO, USA). Antibodies for anti-phospho-ERK-1/2, anti-total ERK-1/2, anti-phospho-p38, anti-total p38, anti-phospho-JNK, anti-total JNK, anti-phospho-IκB, anti-phospho-Akt, anti-Akt, anti-phospho-Src, anti-Src, and anti-Btk were obtained from Cell Signaling Technology Inc. (Beverly, MA, USA). Anti-Phospho-Btk (GTX61791) antibody was obtained from GeneTex (Irvine, CA, USA). Anti-c-Fos, anti-NFATc1, anti-IκB, and anti-PLCγ2 antibodies were purchased from Santa Cruz Biotechnology (Santa Cruz, CA, USA). Anti-Duoxa1 antibody was obtained from Bioss Inc (BS-11433^®^, Wobun, MA, USA). Donkey anti-rabbit and anti-mouse immunoglobulin secondary antibodies were purchased from Enzo Life Sciences (Farmingdale, NY, USA).

### 4.2. Preparation of Mouse BMMs and TRAP Assay

BMCs were isolated from 5-week-old male ICR mice by flushing the femurs and tibias with α-MEM supplemented with 10% FBS and 1% antibiotics [26]. BMCs were cultured on culture dishes in α-MEM supplemented with 10% FBS and M-CSF (10 ng/mL) for 1 day. Then, non-adherent cells were plated to petri dishes and cultured in the presence of M-CSF (30 ng/mL) for 3 days. After the non-adherent cells were washed out, the adherent cells were used as BMMs. To the complete formation of osteoclasts from these BMMs, the cells were seeded in a 48-well plate (3.5 × 10^4^ cells/well) in complete medium containing M-CSF (30 ng/mL) and RANKL (100 ng/mL) and cultured for 3–4 days. BMM-derived MNCs were fixed in 3.7% formalin for 10 min, permeabilized with 0.1% Triton X-100 for 5 min, and then stained with TRAP (Sigma, St. Louis, MO, USA). TRAP-positive MNCs with more than three nuclei were counted as osteoclasts. The size of the osteoclasts was determined by image analysis using the image J software (National Institutes of Health, Bethesda, MD, USA).

### 4.3. Retrovirus-Derived Duoxa1 Gene Transfer and siRNA Transfection in BMMs

The retroviral vector (pMX), pMX-Duoxa1-IRES-EGFP, and mouse negative control (siControl) and Duoxa1 (siDuoxa1) siRNAs were ordered and synthesized by Bioneer Co. (Daejeon, Korea). The transfection assay of retroviral genes and siRNA was performed as previously described [26]. Plat-E retroviral packaging cells were seeded in culture dish before 1 day transfection. The next day, packaging of the retroviral vectors pMX-IRES-EGFP and pMX-Duoxa1-IRES-EGFP was transfected into plat-E cells using X-tremeGENE 9 (Roche, Nutley, NJ, USA) according to the manufacturer’s protocol. After 2 days culture, the culture supernatants of the retrovirus-producing cells were collected. For retroviral infection, non-adherent BMCs were cultured in M-CSF (30 ng/mL) for 2 days. The BMMs were incubated with viral supernatant pMX-IRES-EGFP and pMX-Duoxa1-IRES-EGFP virus-producing plat-E cells together with polybrene (10 µg/mL) and M-CSF (30 ng/mL) for 6 h. After infection, the BMMs were induced to differentiate in the presence of M-CSF (30 ng/mL) and RANKL (100 ng/mL) for 4 days. BMMs were also transfected with 10 nM of siRNA oligonucleotides using Lipofectamine 3000 (Invitrogen, San Diego, CA, USA) according to the manufacturer’s instructions. Briefly, after incubating BMMs in α-MEM containing 10% FBS, siRNA (10 nM)-Lipofectamine 3000 (0.5 µL/48-well or 3 µL/6-well) mixtures in Opti-MEM (Invitrogen, San Diego, CA, USA) were added to BMMs and incubated for 6 h, respectively. The medium was then replaced with fresh complete α-MEM and the cells were further cultured with osteoclastogenic medium.

### 4.4. Actin Polymerization

BMCs were cultured in M-CSF (30 ng/mL) for 2 days. The cells were transfected with the indicated retrovirus or siRNA as described above and further cultured in the presence of M-CSF (30 ng/mL) and RANKL (100 ng/mL) for 3 days. The cells were fixed in 3.7% formalin for 15 min, permeabilized with 0.1% Triton X-100 for 10 min, incubated with 0.25% bovine serum albumin (Sigma, St. Louis, MO, USA) for 30 min, and stained with phalloidin (Life Technologies, Grand Island, NY, USA) and a DAPI solution (Life Technologies) to visualize F-actin and nuclei, respectively. Cell fluorescence was detected by a laser scanning confocal microscope (Olympus FV1000, Olympus Corp., Center Valley, PA, USA). The images were analyzed using the Image-Pro Plus software version 4.0 (Media Cybernetics, Silver Spring, MD, USA). The change in the F-actin ring on mature osteoclasts was quantified by calculating the ratio of actin ring positive (AR+) osteoclasts versus actin ring negative (AR−) osteoclasts. The osteoclasts with normal F-actin rings were considered as AR+ osteoclasts and osteoclasts without or disrupted F-actin rings were considered as AR− osteoclasts. It was defined as disrupted actin rings if less than half of them showed typical morphology of F-actin rings

### 4.5. Bone Resorbing Assay

Mature OCs were prepared from the co-culture of BMCs and primary osteoblasts (OBs) as described previously [26]. Briefly, BMCs (1 × 10^7^ cells) and primary OBs (1 × 10^6^ cells) were incubated in collagen gel-coated culture dishes in the presence of 10^−8^ M VitD_3_ and 10^−6^ M PGE_2_ for 10–12 days. Mature OCs were detached using 0.1% collagenase and re-seeded in dentin slices. After 1 h, the cells were transfected with the indicated retrovirus or siRNA as described above and further cultured in the presence of RANKL (100 ng/mL). The cells re-seeded in dentin slices were completely removed using 10% sodium hypochlorite after 48 h. Dentin slices were stained with hematoxylin to detect resorption pits. The total area of resorbing pits was determined under a microscope and were quantified using Image-Pro Plus version 4.0 (Media Cybernetics, Silver Spring, MD, USA). To confirm the survival of mature OC by retrovirus and siRNA transfection, mature OC replanted in 48-well plates in the same manner as in dentin slices, and after 48 h, stained with TRAP solution.

### 4.6. qRT-PCR

The template first-strand cDNA from 1 µg of total RNA was synthesized using SuperScript II Reverse Transcriptase purchased from Thermo scientific (Thermo Fisher Scientific, Wilmington, DE, USA). The cDNA template amplifications were detected with Accupower green star qRT-PCR master mix using an ExcyclerTM 96 Real-Time Quantitative Thermal block (Bioneer, Daejeon, Korea). The following primers were used for the qRT-PCR: *GAPDH* forward: 5′-TCAAGAAGGTGGTGAAGCAG-3′; reverse: 5′-AGTGGGAGTTGCTGTTGAAGT-3′; *Duoxa1* forward: 5′-CCCACAGGATGCAGCCTCAC-3′; reverse: 5′-ACCGGTAGTGGGGGCTCAAG-3′; *OSCAR* forward: 5′-GGAATGGTCCTCATCTCCTT-3′; reverse: 5′-TCCAGGCAGTCTCTTCAGTTT-3′; *c-Fos* forward: 5′-AGTCCATTTGCTGACCCCAC-3′; reverse: 5′-GGATGGTCGTGTTGATGCG-3′; *NFATc1* forward: 5′-GAGTACACCTTCCAGCACCTT-3′; reverse: 5′-TATGATGTCGGGGAAAGAGA-3′; *CTR* forward: 5′-TCCAACAAGGTGCTTGGGAA-3′; reverse: 5′-CTTGAACTGCGTCCACTGGG-3′; *β_3_-integrin* forward: 5′-GGAGTGGCTGATCCAGATGT-3′; reverse: 5′-TCTGACCATCTTCCCTGTCC-3′; *DC-STAMP* forward: 5′-TCCTCCATGAACAAACAGTTCCA-3′; reverse: 5′-AGACGTGGTTTAGGAATGCAGCTC-3′; *Atp6v0d2* forward: 5′-GACCCTGTGGCACTTTTTGT-3′; reverse: 5′-GTGTTTGAGCTTGGGGAGAA-3′; *CtsK* forward: 5′-CCAGTGGGAGCTATGGAAGA-3′; reverse: 5′-CTCCAGGTTATGGGCAGAGA-3′. The detection of amplification parameters proceeded with initial denaturation at 95 °C for 5 min, and 40 cycles of 3-step PCR: denaturation at 95 °C for 1 min, annealing at 60 °C for 30 s, and the final extension at 72 °C for 1 min. The data were validated by *GAPDH*, and were evaluated as the mean fold change. Relative gene expression was calculated by evaluating qRT-PCR data using the 2^−ΔΔCt^ method.

### 4.7. Western Blotting

After the treatments, cells were rinsed three times with cold phosphate-buffered saline, lysed with lysis buffer, and centrifuged at 10,000× *g* for 15 min. The protein content in the extract was determined using a Bio-Rad DC Protein Assay Kit (Bio-Rad Laboratories Inc., Hercules, CA, USA) and proteins were denatured. Equal amounts of protein (10–20 µg) were separated to sodium dodecyl sulfate-polyacrylamide gel electrophoresis (10% gels), and transferred to polyvinylidene difluoride membranes (Millipore, Bedford, MA, USA). For blocking the non-specific binding on membranes were reacted with 5% nonfat milk in Tris-buffered saline with 0.1% Tween 20 (TBST) for 1 h, and washed with TBST, and incubated with primary antibodies for overnight at 4 °C. The membranes were incubated for 2 h with appropriate secondary antibodies. After washing 3 times with TBST, the target band was detected using an Immobilon Western Chemiluminescent HRP Substrate (Millipore, Billerica, MA, USA). Actin was used for the loading control. Densitometric analysis was performed using Image J software.

### 4.8. ROS Assay

ROS levels were measured by the ROS assay kit, ab113851 (Abcam, Cambridge, MA, USA), following the manufacturer’s protocol. In brief, starved BMMs were incubated with α-MEM lacking phenol red medium and then stored in dark conditions for 10 min with 21, 71-dichlorofluorescein diacetate (DCFH-DA, 10 µM). DCF fluorescence (excitation, 488 nm; emission 515–540 nm) was measured. Confocal fluorescence images were obtained using an Olympus FV-1000 confocal laser scanning microscope (Olympus, Tokyo, Japan).

### 4.9. Statistical Analysis

All data analyses were performed at least three times, and data are indicated with the mean ± standard deviation (SD). Statistical differences were confirmed with one-way or repeated-measures ANOVAs followed by Tukey’s HSD test. A *p* value < 0.05 was defined to indicate a statistically significant difference.

## Supplementary Materials

The following are available online at https://www.mdpi.com/1422-0067/21/17/6416/s1, Figure S1: BMMs were transfected with siControl or siDuoxa1 using lipofectamin3000, and cultured in the presence of M-CSF (30 ng/mL) for 3 days. Cell viability was analyzed by an XTT assay. Figure S2: Overexpression of Duoxa1 increases RANKL-induced ROS production.

## References

[1] Schieber M., Chandel N.S. (2014). ROS Function in Redox Signaling and Oxidative Stress. Curr. Boil..

[2] Bae Y.S., Oh H., Rhee S.G., Yoo Y.D. (2011). Regulation of reactive oxygen species generation in cell signaling. Mol. Cells.

[3] Domazetovic V., Marcucci G., Iantomasi T., Brandi M.L., Vincenzini M.T. (2017). Oxidative stress in bone remodeling: Role of antioxidants. Clin. Cases Miner. Bone Metab..

[4] Filaire E., Toumi H. (2012). Reactive oxygen species and exercise on bone metabolism: Friend or enemy?. Jt. Bone Spine.

[5] Kumar J., Teoh S.L., Das S., Mahakknaukrauh P. (2017). Oxidative Stress in Oral Diseases: Understanding Its Relation with Other Systemic Diseases. Front. Physiol..

[6] Agidigbi T.S., Kim C. (2019). Reactive Oxygen Species in Osteoclast Differentiation and Possible Pharmaceutical Targets of ROS-Mediated Osteoclast Diseases. Int. J. Mol. Sci..

[7] Ponzetti M., Rucci N. (2019). Updates on Osteoimmunology: What’s New on the Cross-Talk Between Bone and Immune System. Front. Endocrinol..

[8] Udagawa N., Takahashi N., Akatsu T., Tanaka H., Sasaki T., Nishihara T., Koga T., Martin T.J., Suda T. (1990). Origin of osteoclasts: Mature monocytes and macrophages are capable of differentiating into osteoclasts under a suitable microenvironment prepared by bone marrow-derived stromal cells. Proc. Natl. Acad. Sci. USA.

[9] Crockett J.C., Mellis D.J., Scott D.G.I., Helfrich M.H. (2010). New knowledge on critical osteoclast formation and activation pathways from study of rare genetic diseases of osteoclasts: Focus on the RANK/RANKL axis. Osteoporos. Int..

[10] Kobayashi N., Kadono Y., Naito A., Matsumoto K., Yamamoto T., Tanaka S., Inoue J.-I. (2001). Segregation of TRAF6-mediated signaling pathways clarifies its role in osteoclastogenesis. EMBO J..

[11] Soysa N.S., Alles N., Aoki K., Ohya K. (2012). Osteoclast formation and differentiation: An overview. J. Med Dent. Sci..

[12] Lee S.-H., Rho J., Jeong D., Sul J.-Y., Kim T., Kim N., Kang J.-S., Miyamoto T., Suda T., Lee S.-K. (2006). v-ATPase V0 subunit d2–deficient mice exhibit impaired osteoclast fusion and increased bone formation. Nat. Med..

[13] De Sandiford S., Kennedy K.A., Xie X., Pickering J.G., Li S. (2014). Dual Oxidase Maturation factor 1 (DUOXA1) overexpression increases reactive oxygen species production and inhibits murine muscle satellite cell differentiation. Cell Commun. Signal..

[14] Seredenina T., Nayernia Z., Sorce S., Maghzal G.J., Filippova A., Ling S.-C., Basset O., Plastre O., Daali Y., Rushing E.J. (2016). Evaluation of NADPH oxidases as drug targets in a mouse model of familial amyotrophic lateral sclerosis. Free. Radic. Boil. Med..

[15] Morand S., Ueyama T., Tsujibe S., Saito N., Korzeniowska A., Leto T.L. (2008). Duox maturation factors form cell surface complexes with Duox affecting the specificity of reactive oxygen species generation. FASEB J..

[16] Fischer H. (2009). Mechanisms and Function of DUOX in Epithelia of the Lung. Antioxid. Redox Signal..

[17] Carvalho D.P., Dupuy C. (2013). Role of the NADPH Oxidases DUOX and NOX4 in Thyroid Oxidative Stress. Eur. Thyroid. J..

[18] Sasaki H., Yamamoto H., Tominaga K., Masuda K., Kawai T., Teshima-Kondo S., Matsuno K., Yabe-Nishimura C., Rokutan K. (2009). Receptor activator of nuclear factor-kappaB ligand-induced mouse osteoclast differentiation is associated with switching between NADPH oxidase homologues. Free Radic. Biol. Med..

[19] Mercer K.E., Sims C.R., Yang C.S., Wynne R.A., Moutos C., Hogue W.R., Lumpkin C.K., Suva L.J., Chen J.R., Badger T.M. (2014). Loss of functional NADPH oxidase 2 protects against alcohol-induced bone resorption in female p47phox^−/−^ mice. Alcohol. Clin. Exp. Res..

[20] Goettsch C., Bábelová A., Trummer O., Erben R.G., Rauner M., Rammelt S., Weissmann N., Weinberger V., Benkhoff S., Kampschulte M. (2013). NADPH oxidase 4 limits bone mass by promoting osteoclastogenesis. J. Clin. Investig..

[21] Shinohara M., Koga T., Okamoto K., Sakaguchi S., Arai K., Yasuda H., Takai T., Kodama T., Morio T., Geha R.S. (2008). Tyrosine Kinases Btk and Tec Regulate Osteoclast Differentiation by Linking RANK and ITAM Signals. Cell.

[22] Lee K., Chung Y.H., Ahn H., Kim H., Rho J., Jeong D. (2016). Selective Regulation of MAPK Signaling Mediates RANKL-dependent Osteoclast Differentiation. Int. J. Boil. Sci..

[23] Song I., Kim J.H., Kim K., Jin H.M., Youn B.U., Kim N. (2009). Regulatory mechanism of NFATc1 in RANKL-induced osteoclast activation. FEBS Lett..

[24] Wei Z.-F., Tong B., Xia Y.-F., Lü Q., Chou G.-X., Wang Z.-T., Dai Y. (2013). Norisoboldine Suppresses Osteoclast Differentiation through Preventing the Accumulation of TRAF6-TAK1 Complexes and Activation of MAPKs/NF-κB/c-Fos/NFATc1 Pathways. PLoS ONE.

[25] Asagiri M., Takayanagi H. (2007). The molecular understanding of osteoclast differentiation. Bone.

[26] Baek J.M., Cheon Y.-H., Kwak S.C., Jun H.Y., Yoon K.-H., Lee M.S., Kim J.-Y. (2018). Claudin 11 regulates bone homeostasis via bidirectional EphB4-EphrinB2 signaling. Exp. Mol. Med..

