# Supplementary material for: Dual Oxidase Maturation Factor 1 Positively Regulates RANKL-Induced Osteoclastogenesis via Activating Reactive Oxygen Species and TRAF6-Mediated Signaling
Source: Int J Mol Sci. 2020 Sep 3;21(17):6416. doi: 10.3390/ijms21176416 (PMC7503776; doi:10.3390/ijms21176416)
Supplement: Supplementary file 1 [file ijms-21-06416-s001.pdf]

Supplementary Figure legends

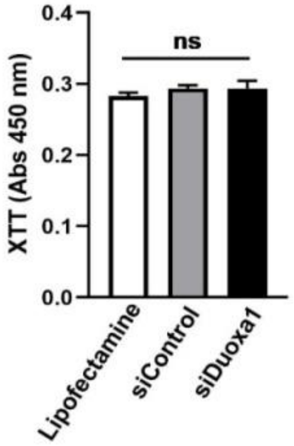

Supplementary Figure 1: BMMs were transfected with siControl or siDuoxa1 using lipofectamin3000, and cultured in the presence of M-CSF (30 ng/mL) for 3 days. Cell viability was analyzed by an XTT assay.

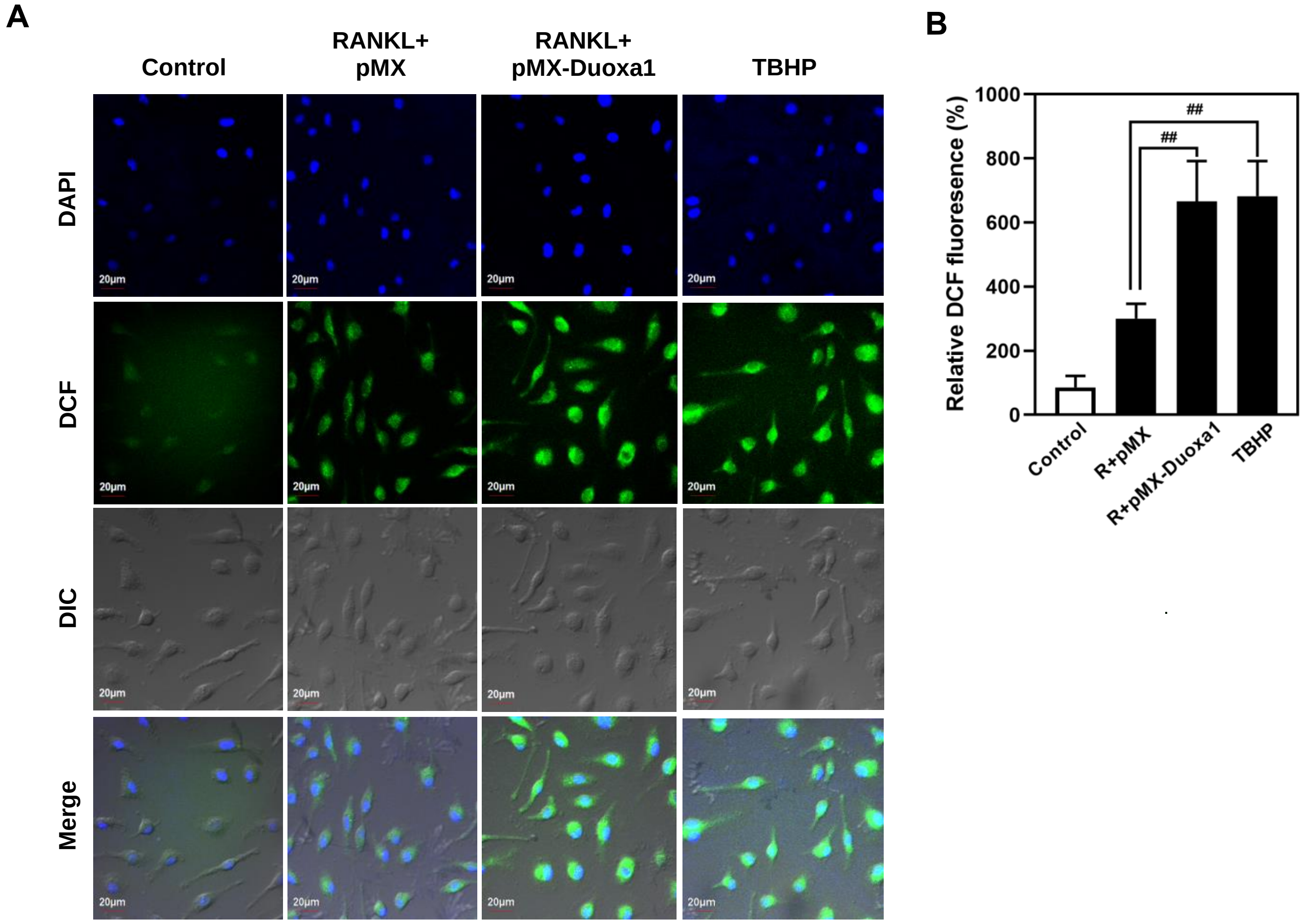

Supplementary Figure 2: Overexpression of Duoxa1 increases RANKL-induced ROS production. **(A)** BMMs transduced with the pMX or pMX-Duoxa1 retrovirus were treated with RANKL for 10 min, and

ROS levels were determined by DCF fluorescence detection using a confocal laser-scanning microscope. **(B)** The DCF fluorescence intensity was confirmed for the region of interest (ROI). Results are representative of at least 3 independent sets of similar experiments. TBHP was used as the standard control for ROS production. Data are presented as the mean ± SD of three independent experiments. $^{\#\#}p < 0.01$ versus the positive pMX.
